# Frontiers in Neurogenesis

**DOI:** 10.3390/cells11223567

**Published:** 2022-11-11

**Authors:** Andreia Vaz, Inês Ribeiro, Luísa Pinto

**Affiliations:** 1 Life and Health Sciences Research Institute (ICVS), School of Medicine, University of Minho, Campus de Gualtar, 4710-057 Braga, Portugal; 2 ICVS/3B’s–PT Government Associate Laboratory, 4710-057 Braga, Portugal; 3 Bn’ML, Behavioral and Molecular Lab, Campus de Gualtar, 4710-057 Braga, Portugal

One of the most intriguing dogmas in neurosciences—the empirical lack of brain neuronal regeneration in adulthood onwards to late life—began to be debunked initially by research groups focused on understanding postnatal (early days/weeks of murine and guinea pigs) neurodevelopmental and neuroplastic events. These early studies were perceived as too provocative and dubious because of the lack of understanding of the extent to which “newly generated cells” differentiate into glia or neurons. For this reason, some members of the scientific community rejected these studies; soon after, with the introduction of _3_H-thymidine autoradiography that specifically targeted the DNA of dividing cells, researchers were able to begin to track down cell proliferation in the adult brain. Interestingly, in the early 1960s, Joseph Altman’s team provided evidence of important plastic events occurring in regions of the young and adult mammalian brains—the dentate gyrus (DG), the neocortex, and the olfactory bulb—and their importance in the regulation of memory and learning. His team’s achievements were welcomed with a lot of scepticism, mostly because of the lack of credibility and immaturity of the early specific neuronal markers used for immunohistochemistry assays and _3_H^−^thymidine labeling on thick brain slices, which raised doubts over the cellular phenotype of these cells—were they neurons or glia? [1,2,3]. Later on, other researchers continued to carry out studies on different species to prove the existence of active brain areas where neurogenesis occurs in adulthood, but were completely proven only in the 1990s with the advent of methodological and technical advancements. The replacement of _3_H^−^thymidine by BrdU (a synthetic analogue of thymidine) was a turning point, as it is taken up during the S-phase of mitosis and tracks down the proliferation of cells. Fred Gage’s team was the first to use BrdU and applied stereology to elucidate the differentiation of neural progenitor cells under normal circumstances and in an enriched environment. Indeed, they found that newborn cells phenotypically identical to granule cells from an adult rat expressed NeuN (back then it was a novel marker that targeted postmitotic neurons) as well as calbindin-D_28k_ (a calcium-binding protein frequently expressed by mature granule cells) and arose from neural progenitor cells (Figure 1). Apart from these findings, they were able to detect the decay of adult neurogenesis in the DG as a consequence of aging [4].

The tenacious curiosity of neuroscientists has taken the field further, with the first proof of evidence of adult neurogenic activity in humans being presented back in 1998. Persons diagnosed with cancer (n = 5) received intravenous infusions of BrdU that revealed plasticity events in the DG—newly generated cells survived and underwent differentiation processes to generate cells phenotypically identical to neurons [7]. More studies have followed in these footsteps, and the discovery of neural markers such as NSE, MAP-2, TUJ-1, O4, GFAP, and S100ß heightened the relevance of the findings that not only supported the role of the DG as an important neurogenic niche in the adult brain (areas whose special microenvironment is enriched with neural stem cells at different stages of commitment) but also its pertinence for important cognitive tasks and emotional processing, which are intertwined in complex, dynamic neuronal networks [8,9,10,11,12,13,14]. Neurogenic niches are not restricted to the subgranular zone (SGZ) of the DG, and, over the years, researchers have been identifying other areas where neurogenesis is active in the adult brain: the subventricular zone (“canonical” niche), hypothalamus, striatum, substantia nigra, cortex, and amygdala. However, it remains to be fully elucidated whether neural stem cells and progenitor cells exclusively originate from the subventricular zone and migrate to these other areas or if they are found in these regions and undergo differentiation processes and become integrated into attracting more attention. The scrutiny of the neurogenic pool in these regions has provided important insights into the regulation of mechanisms and processes. Briefly, throughout the neurogenic process in the adult hippocampus, newborn neurons go through a short migration route from the SGZ to the DG granule cell layer, where they will be permanently integrated into the existing neuronal circuitry. These processes occur in four phases (thoroughly reviewed by the authors of [5]) and are regulated by a multitude of factors, which include transcription factors and signaling pathways (e.g., SOX-2, Wnt signaling, and NeuroD1); brain-derived neurotrophic factor; the activation of the hypothalamus–pituitary–adrenal axis, which elevates the levels of glucocorticoids in the blood and downregulates neurogenesis; proinflammatory molecules (e.g., TNF-α and IL-1β); and physical activity (enhances the expression of neurotrophic factors). However, these intrinsic and/or extrinsic regulatory factors are promoters and suppressors of neurogenesis, and are dependent on the individual context, physiology, pathology, behavior, and resilience and/or susceptibility to environmental cues (e.g., chronic stress), which downregulate or upregulate adult neurogenesis, affecting cell proliferation, neuronal differentiation, and cell survival [5,15,16,17] (Figure 1).

Inevitably, the continuous efforts to understand the whole process, from the proliferation and fate specification/commitment of adult neural progenitors to the morphogenesis, migration, axon/dendritic development, synapse formation, and integration of new neurons into the existing circuitry, have provided invaluable knowledge on the role of these newly generated neurons, and of the abnormalities occurring in their generation, in brain functions, such as cognition, learning, memory, and mood regulation. Over the years, researchers have become less focused on neurocentric research and have been looking for the influence of other neural cells on the maintenance of homeostasis in health and disease. This is a particular hallmark because glial cells are more predominant than neurons and responsible for the genesis and maintenance of circuits as well as neural specification, and the neuron–glia crosstalk influences the integrity of circuits and neurodevelopment. In the latter, as the cortex development rises from an early pseudostratified neuroepithelium that originates radial glia—the neural stem cells that generate neurons—initially through direct neurogenesis, it then enters a gliogenic phase to generate oligodendrocytes (OLs) and astrocytes [18,19,20,21,22]. Furthermore, microglia have an important regulatory role by secreting mitogenic factors, regulating hippocampal neurogenesis by pruning supernumerary neurons, clearing cellular and axon debris, and, together with astrocytes and OLs, generating and regulating synaptic function as well as neurotransmitter turnover whilst providing metabolic support [23]. These cells are all intertwined and critical for brain homeostasis in either disease substrates or in the absence of diseases. For this reason, a growing number of research groups are becoming very interested in exploring the extent to which glial cells are newly born in the adult brain, but their repercussions on mood, behavior, and cognitive performance as well as functioning are still not very clear [24]. Indeed, adult-born neurons in the DG undergo structural and functional plasticity maturation in the trisynaptic hippocampal circuit, which is extremely important for hippocampus-dependent functions, more precisely for encoding memories and mood regulation. It is not at all surprising that many neurological, neurodevelopmental, and neuropsychiatric diseases are the consequence of injurious functional, structural, and cellular changes in the brain triggered by internal and external factors such as psychological stress, age-dependent cognitive decline, mood disorders (e.g., major depressive disorder), Alzheimer’s disease, Parkinson’s disease, epilepsy, autism spectrum disorder, schizophrenia, and cocaine addiction [25,26,27,28,29,30]. Mounting evidence has consecutively proven that some drugs, such as clozapine (an atypical antipsychotic drug) and antidepressants, can rescue neuroplasticity events that culminate in the increased generation of adult-born neurons and recovery from a depressive-like phenotype in animal models of depression where neuro- and glioplastic maladaptations often result in the manifestation of pathological traits, from which depressive behavior is a paradigmatic example [31,32]. For instance, among humans studies have reported that persons diagnosed with major depressive disorder, without being enrolled in pharmacological treatment, have smaller hippocampal volume and reduced glial as well as neuronal cell size and populations compared to persons without a psychiatric diagnosis [33].

Nevertheless, biomedical research has relied mostly on in vivo preclinical models for decades, and these models have undeniably advanced the knowledge on the onset, progression, and chronicity of diseases as well as drug development. To overcome the need to rely keenly on human tissue from more invasive biopsies, autopsies, or animal models, alternative strategies continue to be devised. Among these, pluripotent stem cells, which have an intrinsic ability to self-renew and differentiate into cell types from the three embryonic germ layers, enable the study of genome–phenotype interactions and the molecular as well as cellular events that occur during these processes. Most importantly, human-induced pluripotent stem cells (iPSCs) recapitulate the human genetic and epigenetic landscape that is absent in animal models, circumventing in this way the limitations of accessing human tissue, which helps to identify the developmental stage where genetic changes emerge, for example, through large-scale genomic analyses [34,35]. iPSC-based disease models are demanding and challenging, yet very attractive tools that have been employed to understand how the distinctive human epigenetic and genetic profiles confer resilience or heightened risk to certain diseases. This way, it is possible to identify risk variants in specific cell types and the degree to which they impact disease-associated interactions between cell types. Ultimately, iPSC-based disease models have the potential to meet the growing dire need to understand and offer more personalized solutions to the clinical and biological/physiological heterogeneity found, for example, in neuropsychiatric diseases [36] (Figure 2).

Understanding the role of novel genes and cytogenic regulators, as well as better dissecting their impact throughout developmental periods and in different behavioral domains, is of paramount importance for increasing our current comprehension of this topic. This Special Issue of the journal *Cells*—Frontiers in Neurogenesis—compiles unique, exciting, and groundbreaking research presented in 17 papers that aim to bring clarity to these events and diseases, and are grouped into four major themes: (1) neurodevelopment; (2) cytogenesis; (3) adult neurogenesis; and (4) the impact of cellular plasticity on neurological and psychiatric disorders.

In the first group, this Special Issue begins with a review by Kristofova M. et al. that explores the endeavours performed to date to understand the influence of MCPH genes on human primary microcephaly (MCPH). These genes are known to be implicated in various molecular and cellular processes, linked to the aetiology of MCPH. The primary interest of this review is the MCPH1 (primary microcephaly type 1) gene, which influences many cellular functions that regulate the division mode of neural stem cells during embryonic brain development. The impairment of this gene has profound consequences for the generation of neurons and self-renewal divisions. The authors provide a comprehensive and complete perspective on the preclinical models employed to date as well as on the consequences on a systemic level in the hope of advancing the field [43]. The following work by Roll L. et al. is grounded in human iPSCs and the generation of cerebral organoids. The team focused on extracellular matrix molecules and looked at the expression patterns of the LewisX trisaccharide motif and the sulfation-dependent DSD-1 chondroitin sulfate glycosaminoglycan epitope in these organoids. They suggest that a differential glycoepitope expression may have a specific role in the early steps of the development of the human central nervous system [44]. This group of articles finishes with neurodevelopmental research performed on xenopus embryos. Kumar V. et al. found that Foxd4l1.1 inhibits chordin (*chrd*) expression during the neuroectoderm formation of Xenopus embryos. *Chrd* physically interacts with bone morphogenetic proteins and inhibits BMP signaling, which triggers the expression of neural-specific transcription factors, including Foxd4l1.1. The mechanisms by which Foxd4l1.1 inhibits *chrd* expression during neuroectoderm formation can be explained as follows: First, Foxd4l1.1 directly binds to FREs (Foxd4l1.1 response elements) within the *chrd* promoter region to inhibit transcription. Second, Foxd4l1.1 physically interacts with Smad2 and Smad3, and this interaction blocks Smad2 and Smad3 binding to activin response elements within the *chrd* promoter. The site-directed mutagenesis of FREs within the *chrd* (−2250) promoter completely abolishes the repressor activity of Foxd4l1.1. With this work, the authors contribute to a better understanding of the tightly regulated mechanism of neural fate acquisition in vertebrate embryos [45].

The second group of articles includes a work that investigates the therapeutic effect of L-thyroxine (L-T4) on vestibular compensation. Briefly, thyroid hormone (TH) signalling governs key processes of cytogenesis and thyroxine, or triiodothyronine, that have been associated with the increased expression of brain-derived neurotrophic factor. With this in mind, Rastoldo G. et al. have undertaken this research on a rat model of unilateral vestibular neurectomy and found that short-term L-T4 treatment reduced vestibular syndrome, significantly promoted vestibular compensation, and promoted microglial rather than neuronal differentiation. In sum, this work brings new insights into alternative pharmacological approaches targeting pathologies such as acute peripheral vestibulopathy or Ménière’s disease [46]. Because the neurobiological correlates in acute peripheral vestibulopathy remain unknown, Marouane E. et al. decided to explore the behavioral and cellular consequences of a vestibular rehabilitation protocol adapted to a rat model of unilateral vestibular neurectomy. They developed a progressive sensory–motor rehabilitation task and quantified the behavioral consequences with a weight distribution device that provides a precise and ecological analysis of posturolocomotor vestibular deficits. Their results highlight that vestibular rehabilitation induces faster recovery of posturolocomotor deficits during vestibular compensation that are associated with a decrease in neurogenesis and an increase in microgliogenesis in the deafferented medial vestibular nucleus. This research provides novel insights into the underlying adaptive neuroplasticity mechanisms of vestibular rehabilitation [47].

As aforementioned, gliogenesis is still enigmatic and has attracted a lot of attention. Machado-Santos A.R. et al. studied the modulation of astrocytes and adult astrogliogenesis in the DG of rats exposed to an unpredictable chronic mild stress protocol, untreated and treated for two weeks with the antidepressants fluoxetine (a selective serotonin reuptake inhibitor) and imipramine (a tricyclic antidepressant). The team found that adult astrogliogenesis in the DG is modulated by stress and imipramine. In addition, the distinct classes of antidepressants used in this study had differential impacts on the astrogliogenic process, which points towards different cellular mechanisms relevant to the recovery from behavioral deficits induced by chronic stress exposure. As such, in addition to those residents, the newborn astrocytes in the hippocampal DG might also be promising therapeutic targets for neuropsychiatric diseases [48]. Interestingly, the adult subventricular zone also retains the intrinsic ability to generate glial cells, namely OLs, through a process called oligodendrogenesis. Changes in brain dynamics occur in the aging brain, and, among these, failures in repair mechanisms, more precisely the decreased regenerative capacity of oligodendrocyte progenitor cells (OPCs) and the consequent loss of OLs as well as myelin, are implicated in the pathogenesis of multiple sclerosis, stroke, and Alzheimer’s disease. Butt A.M. et al. provide a review covering the events in the subventricular zone leading to the replenishment of OPCs and therefore promoting the remyelination of the aging brain; the main differences in OLs’ distinct sources and their responses to demyelination highlighted the main applications for regenerative therapies in the aging brain [49].

The third group of articles in this Special Issue focuses on adult neurogenesis. As described previously, there are neural stem cells in the brain that give rise to adult-born neurons through a process called adult neurogenesis. This process is affected by age and neurodegenerative diseases, promoting a notable drop in adult neurogenesis. Alzheimer’s disease is one of the most common neurodegenerative diseases with alterations in neurogenesis, which correlate with disease severity; however, the reason for this impairment remains uncertain. Several key factors are well-known to play a role in this disease, but how this disease state alters neurogenesis is lacking comprehension. Fortunately, relevant research has been carried out, and Essa H. et al. have updated the most recent approaches to neurogenesis in Alzheimer’s disease as well as revised improvements in the therapeutic procedures that are being studied [50]. Similar to what happens in Alzheimer’s disease, where the hippocampus suffers atrophy and neurogenesis is clearly impaired, post-traumatic stress disorder patients usually present with the same pathological phenotype. A study conducted by Willinger and Turgeman focused on the role of a specific proinflammatory cytokine, interleukin-17A, and found that it modulates neurogenesis following exposure to stress. They also conclude that IL-17 may be important for sustaining social behaviors, as increased levels of this cytokine prevented social deficits in trauma-exposed mice [51].

Hypertension is a risk factor for cognitive decline, and aging increases the risk of memory loss. Recent research points to the beneficial effects that the use of antihypertensives can provide to patients concerning dementia prevention. Yoo S. et al. aimed to clarify how the combined use of antihypertensives and statins (blood-cholesterol-lowering drugs that are also used as preventive measures for cardiovascular events; HMG-CoA reductase inhibitors) has an impact on cognitive decline. They examined the effects of the combined use of atorvastatin (statin) and captopril (antihypertensive; angiotensin-converting enzyme inhibitor) on memory function, anxiety-like behavior, adult hippocampal neurogenesis, and angiogenesis in middle-aged mice. Their findings suggest that statin and antihypertensive treatment may decrease anxiety, which is of particular importance for patients that have impairments in the extinction of aversive memories, such as post-traumatic stress disorder [52]. Besides age and neurodegenerative diseases, thyroid hormones (THs) are also classically reported as factors affecting adult neurogenesis. Most recently, the effects that THs exert on the mammalian brain have been uncovered. The work from Mayerl S. et al. sheds a light on this topic, highlighting those distinct actions of TH transporters (Mct8 and Oatp1c1) that are needed at multiple steps to ensure appropriate adult hippocampal neurogenesis. According to their work, the lack of Oatp1c1 resulted in increased neuroblasts and reduced immature neurons, and that Mct8 impacts neuron formation [53]. Galectins are evolutionarily conserved proteins also very relevant in disease. Galectin-3 (Gal-3) is a multifunctional protein that leads to inflammation in disease; however, little is known about its role in the central nervous system, as it is more often studied from an immunological point of view. Soares L. et al. wrote a complete revision about the role of Gal-3 in the central nervous system, highlighting its relevance in the context of Alzheimer’s disease and in a variety of brain injuries, among other diseases. This protein has surprisingly specific functions in regulating adult subventricular zone neurogenesis in disease. In this review, the authors highlight remarkable effects of Gal-3 on brain pathology and adult neurogenesis [54]. In fact, adult neurogenesis can be impaired by many diseases, and there is an important system that participates in its control. The mammalian circadian system controls a variety of body and brain functions as well as physiological processes, including adult neurogenesis. This modulation can be achieved via neurotransmitters, hormones, and neurotrophic factors that will be crucial for brain plasticity. Ali and von Gall provided a detailed review that helps to understand the role of the circadian system in adult neurogenesis modulation from two points of view: the systemic and cellular levels. A better understanding of the circadian system can be valuable in developing new treatment strategies for chronodisruptive and neurodegenerative diseases [55].

Neurogenesis in mammals occurs during embryonic and postnatal development. However, in eutherians and marsupials, part of brain development occurs after birth. Bartkowska K. et al. review the advances that are being made in understanding the postnatal neurogenesis that occurs postnatally in marsupials and eutherians. They highlight the study of the DG and olfactory bulb in marsupial and eutherian species, as these are the prime regions where neurogenesis is known to occur. Nevertheless, the authors do not end without emphasizing the existence of a new brain region where immature neurons exist, namely the piriform cortex, which has been increasingly explored in the field of postnatal neurogenesis [56].

At last, this Special Issue presents a fourth group of articles on the impact of cellular plasticity on neurological and psychiatric disorders. G-protein-coupled receptors (GPCRs) play an important role in the neurobiology of psychiatric disorders. Monfared R.V. et al. found that GPCRs were overrepresented and found to be dysregulated in four psychiatric disorders, namely autism spectrum disorder, schizophrenia, bipolar disorder, and major depressive disorder. The authors also correlated the age-associated expression of GPCRs with the tendency to be dysregulated in the four presented psychiatric disorders. They further found that autism spectrum disorder had a greater tendency to be age-associated with GPCR dysregulation. This study suggests that targeting GPCRs could serve as a common therapeutic strategy to treat some clinical symptoms crosswise psychiatric disorders [57]. Knowing that methamphetamine is highly recognized for its addictive potential, and that its use is associated with important neurological changes that result in psychosis, altered cognitive function, anxiety, and depression, among others, Bravo J. et al. decided to explore how neurons affect microglia activation under methamphetamine exposure. Evidence has pointed to the required interplay between neurons and microglia to cause addiction. The authors cocultured microglia with primary neuronal cells under the effect of methamphetamine and discovered that microglia activation can be partially prevented by neurons via astrocytes. This interaction seems to be achieved by increasing arginase 1 expression and strengthening the CD200/CD200r pathway. They also detected an increase in the pre- and postsynaptic individual areas, suggesting improvement in neuronal plasticity. This study demonstrates that the interplay between neurons and microglia by contact-dependent mechanisms can attenuate proinflammatory events, namely methamphetamine-induced microglial activation [58].

Given that the mutual involvement of expression and methylation dynamics on genome regulation can have a great impact on various disorders, including psychological disorders and cancer, Loeffler-Wirth H. et al. performed a joint study on the gene expression and DNA methylation of the brain across the human lifespan. They aimed to describe changes in cellular programs and their regulation by epigenetic mechanisms from babies to elderly adults. Interestingly, the authors observed an accumulation of methylation in bivalent genomic regions with age, which might have an important function in cell differentiation and development, as well as the closing off of these developmental pathways. In fact, this accumulation might be relevant for the proper homeostasis and functioning of the adult brain, including a balanced control of neural stem/progenitor cell self-renewal, adult neurogenesis, repair, learning, and memory, as they require a residual level of developmental plasticity. By comparing aging profiles of the healthy brain with expression and methylation data of brain lower-grade gliomas, they also found that glioblastoma-like and astrocytoma-like tumors have higher cellular plasticity in the developing healthy brain, while oligodendrogliomas have more stable differentiation more reminiscent of the aged brain. This study highlights the relevance of performing large-scale analyses and increasing cellular resolution to link molecular players such as gene expression and DNA methylation with neuronal nets and cognitive functions in addition to their development and aging [59].

Building upon advances in cellular, molecular, behavioral, and computational neurosciences, adult brain cytogenesis will become even clearer. These avenues will push the field further toward the understanding of the influence of internal and external stimuli on individual neural cells, their myriad of roles across the lifespan, and the circuitry dynamics underlying structural plasticity, which is pivotal in health and disease.

## Figures and Tables

**Figure 1:**
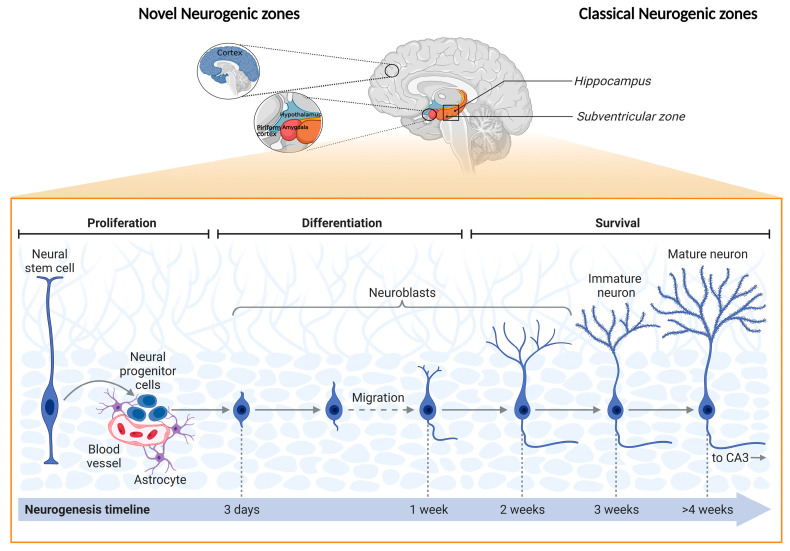
Schematic representation of “novel” and classical adult neurogenic zones, as well as their main cellular stages. Representation of the source of progenitor cells in different brain regions: the “novel” adult neurogenic zones—the prefrontal cortex, hypothalamus, striatum, amygdala, and piriform cortex—and the prime adult neurogenic zones—the hippocampus and subventricular zone (SVZ) [5]. The generation of new neurons has been extensively studied in the hippocampal dentate gyrus (DG) and the SVZ. In the DG, progenitor cells located in the subgranular zone (SGZ) divide and give rise to quiescent radial glial cells (neural stem cells) that can commit to neural progenitor cells and mature into neurons. These neurons will migrate to the granule cell layer to integrate into existing hippocampal circuitries. Experimental evidence has proposed that progenitor cells can deviate from the normal route in the DG and SVZ and differentiate as well as maturate in different brain regions, namely the prefrontal cortex, striatum, substantia nigra, and amygdala, considered the “novel neurogenic zones” [5,6]. Figure created with BioRender.com (Accessed on 25 October 2022).

**Figure 2:**
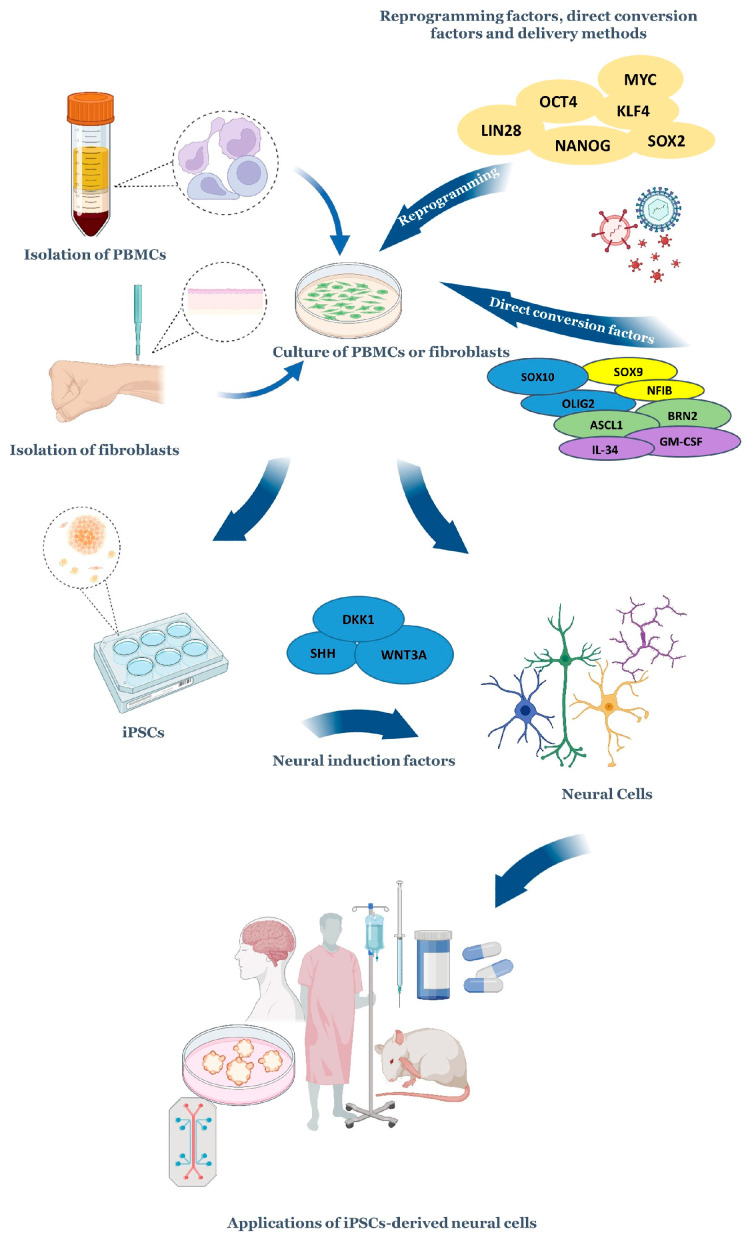
A summary of the process of obtaining and exploiting iPSC-derived neural cells. Somatic cells, such as peripheral blood mononuclear cells and fibroblasts, are typically collected from persons, as these cells are easy and not excessively painful to obtain. After their collection, these cells have different fates: (1) They can be reprogrammed into iPSCs through delivery strategies that include episomal vectors, non-integrating viral vectors, transient DNA transfection, transposons, and protein transduction. Therefore, these cells are forced to express specific transcription factors that reprogram the cells into a pluripotent state (e.g., SOX2, OCT4, and others highlighted in the image). The exposure to lineage-specific neural induction factors promotes the differentiation into neural cells. (2) They can be induced into neural cells in a process called transdifferentiation. This can be achieved through the overexpression of transcription factors, miRNAs, and exposure to chemical cocktails/direct conversion factors that mimic the signaling environment of the developing brain (growth factors and other signaling molecules [37,38]). Depending on the study’s needs, neural cells can be attained in 2D models or more complex 3D models that include brain organoids alone or connected to other organoids on platforms commonly known as organoids-on-a-chip, which can aid drug discovery and evaluate drug efficacy as well as some parameters, including toxicity. Several teams have transplanted iPSC-derived neuronal cells into the brains of mice to understand the peculiarities of circuit dynamics, and humans have already received iPSC-derived cellular transplants [39,40,41]. Adapted from [42]. Figure created with Microsoft PowerPoint^®^ (2016) BioRender.com (Accessed on 25 October 2022).
